# Mocking Bird ( *Mimus polyglottos* ) calls potentially confound acoustic indices of bird diversity and provide a potential heuristic to distinguish them

**DOI:** 10.17912/micropub.biology.001148

**Published:** 2024-07-09

**Authors:** Barbara Kalta, Andrew Gregory

**Affiliations:** 1 Biology , University of North TX; 2 Biology , University of North Texas, Denton, Texas, United States

## Abstract

This study explores the increasing use of autonomous recording units (ARUs) in wildlife surveys. While ARUs offer cost-effective and efficient data collection, challenges arise in analyzing large datasets and accurately assessing species abundance. Our research focuses on avian communities, emphasizing the impact of vocal mimicry by Northern Mockingbirds ( *Mimus polyglottos* ) on survey accuracy. Utilizing the Merlin Bird ID application, we found an average accuracy rate of ~81.3%, with mockingbirds contributing ~31% of false positive identifications. Finding potential solutions for distinguishing mimics in bioacoustic survey data is crucial for enhancing accuracy as researchers increasingly adopt this methodology in the future.

**Figure 1. Results from our initial acoustic analysis and application accuracy analysis:**
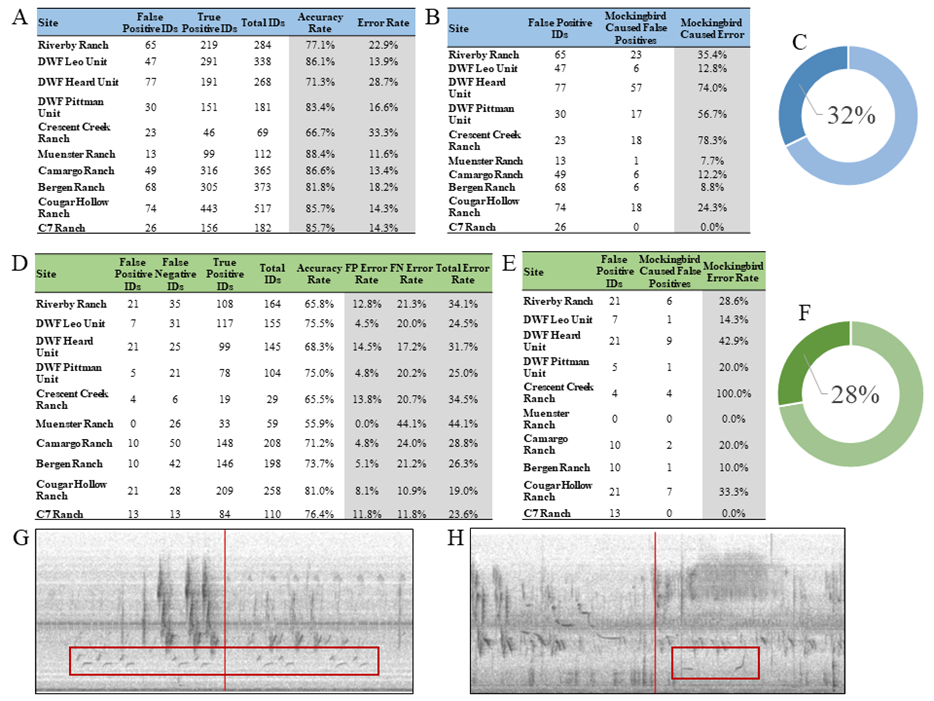
**(A)** Initial acoustic analysis results for our ten sites. True positives and false positives identified by the Merlin Bird ID application in each recording were documented, and accuracy and error rates calculated. This initial acoustic analysis resulted in an average error rate of ~19%. **(B)** Number of false positives in our initial acoustic analysis caused by Northern Mockingbirds ( *Mimus polyglottos* ), out of the total number of false positives identified. **(C)** Percentage of total false positives identified in the initial acoustic analysis that were caused by Northern Mockingbirds. **(D)** Application accuracy analysis results for our ten sites. True positives, false positives, and false negatives identified by the Merlin Bird ID application and researcher in each recording were recorded. This accuracy analysis resulted in an average error rate of ~29%, with the false positive error rate averaging at ~8% and the false negative error rate averaging at ~21%. **(E)** Number of false positives in our accuracy analysis caused by Northern Mockingbirds ( *Mimus polyglottos* ), out of the total number of false positives identified. **(F)** Percentage of total false positives identified in the accuracy analysis that were caused by Northern Mockingbirds ( *Mimus polyglottos* ). **(G)** Spectrograph example of a Northern Mockingbird ( *Mimus polyglottos* ) mimicking a Northern Bobwhite ( *Colinus virginianus* ; red box). Northern Mockingbirds typically mimic other species’ songs two to four times in a row, then may switch to another species’ song. In this example, the Northern Mockingbird mimics the Northern Bobwhite in a series of two to three calls in immediate and rapid succession. In this recording, the Merlin Bird ID application identified positively that a Northern Mockingbird was present, but also falsely identified the presence of a Northern Bobwhite. **(H)** Spectrograph example of a true positive Northern Bobwhite song ( *Colinus virginianus* ; red box). Note that the actual Northern Bobwhite call there is a single two trill call--the stereotypically “ *bob-white* ” intonation. This tendency of Mockingbirds to duplicate calls in rapid succession created a useful heuristic for us to use to rapidly identify likely Mockingbird calls driving false identifications in our dataset and correct them to increase the overall accuracy of our acoustic surveys.

## Description

Bird species richness and diversity at a site are often used as a proxy to understand overall system health and naturalness; however, collection of these data can be time consuming and require specialized knowledge (Albuquerque and Gregory 2017; Gregory et al. 2021) . As costs go down and technology improves, bioacoustical surveys of wildlife are becoming an increasingly popular survey practice (Willacy et al. 2015; Frommolt 2017) . Using autonomous recording units (ARUs) for wildlife surveys allow scientists to collect large quantities of data with little effort, compared to other more labor-intensive methods of field observations (Priyadarshani et al. 2018) . Using ARUs also allows researchers to conduct surveys without the need for a highly trained and specialize work force present in the field, which can reduce costs, increase detection reliability, and decrease inter-observer bias (Priyadarshami et al. 2018). Additionally, the use of ARU’s reduces the need to have an observer present in the field conducting the observations and potentially altering the behavior being observed (Brandes 2008) . However, like all new and emerging technologies and methodological advancements, there are some drawbacks to bioacoustical surveys.

Specifically, while wildlife presence and occupancy may be established using the data collected from ARUs, abundance and density of species may be more difficult to assess due to the lack of a visual component or ability to identify individual signalers in the collected dataset (Perez-Granados 2019) . Additionally, collection of information using ARUs can result in large datasets, which can be incredibly time consuming and labor intensive to clean, code, and analyze to extract meaningful trends (Priyadarshani et al. 2018) . New technologies, such as the development of Artificial Intelligence (AI), are able to overcome some of these limitations by assisting in data cleaning and analysis (Sharma et al. 2023) , but trained human observers are still more proficient at identifying misclassifications in the datasets.

One common use of ARUs has been for research assessing avian species occupancy and community composition at a site. One of the main forms of communication in birds is sound, making bioacoustics an appealing method for surveying them (Kumar 2003) . Moreover, *most* of the calls and songs bird’s produce are species specific and distinct, allowing for accurate identification of presence and community composition from ARU survey data. However, there are some specialist species of birds that mimic other species-specific vocalizations, which may confound and bias acoustical survey data. One such mimic is the Northern Mockingbird ( *Mimus polyglottos* ). The Northern Mockingbird (hereafter mockingbird) is found throughout the continental United States (Derrickson et al. 1992) . Male mockingbirds have been observed to mimic the repertoire of up to 150 different species’ songs, although this may increase as the male ages (Derrickson et al. 1992) . Such mockingbird vocal mimicry may trick both researchers and AI’s into misidentifying a mockingbird song as the song of the species’ that the mockingbird is mimicking; thereby, biasing acoustical survey data. When observers are in the field and acoustic data is coupled with visual observations, this bias can be accounted for, however as more researchers turn to passive bioacoustical monitoring for assessing bird communities, the presence and prevalence of mimics such as the mockingbird can become increasingly problematic.

We collected acoustic data across ten ranches in North Texas, four within Cooke County, two within Clay County, one within Fannin County, one within Parker County, one within Hill County, and the last within Denton County. Each ranch had between one to ten acoustic monitors set up within the ranch boundaries, with the number of monitors used selected to sample each ranch at equal intensity (one monitor per 50.6-57.8 ha). For acoustical surveys we used the Wildlife Acoustics Song Meter Micro passive acoustic monitors. We distanced acoustic monitors at least 700 meters apart in order to avoid sensors sampling the same acoustical space on the landscape. Monitors were programmed to record for one hour/day, starting a half hour before sunrise. Acoustic monitoring occurred for five consecutive days, resulting in five hours of acoustic data collected from each monitor, and we sampled each location twice during the season. We randomly selected two 30-minute recordings from each monitoring session for detailed coding and analysis.

To analyze our acoustic data, we utilized the publicly sourced bird identification application, Cornell Lab’s Merlin Bird ID (Cornell University (Version 2.1.5)). We took each 30-minute recording and ran it through the application, then confirmed each species detection as either a true positive (a detection confirmed by a researcher) or false positive detection (a detection rejected by a researcher). In addition, we also completed an accuracy assessment for the Merlin Bird ID app bird detections for each site, and corrected our detections to at least 80% accuracy. We ran three-minute increments through the application while a researcher was simultaneously identifying any species heard. With this analysis we were able to confirm all true positives, false positives, and false negatives detections (an identification made by a researcher, but not detected by the Merlin Bird ID app) of bird species. These statistics allowed us to assess the accuracy and error rates associated with using the Merlin Bird ID application. In addition to accuracy and error rates, we also assessed the ability of the Merlin Bird ID app to repeatably provide the same bird identifications within a single survey period. To accomplish this, we randomly selected 20, five minute segments of recordings, and repeatedly analyzed them four times (400 total minutes), with a freshly downloaded and untrained version of the Merlin Bird ID app. We kept track of the species richness obtained from each survey and the number of times each call was heard, and ranked the calls heard form most common to least common in each session. We further estimated the number of false positive detections that were caused specifically by mockingbirds.

To identify the effects of mockingbird calls on our bird ID accuracies, we used a heuristic rule and identified any instances in the acoustic data where the same bird called in rapid succession (generally <2 seconds pause between calls), followed immediately or interrupted by a call of a different species as a putative mockingbird call. We used this heuristic to distinguish potential mockingbird calls from those of the target species being mimicked. In addition, mockingbirds can potentially help identify the occurrence of rare or cryptic birds on the landscape, as mockingbirds are quite common, but can only mimick what they have previously heard. Thus, if a mockingbird is making the sound of an otherwise undetected bird, you can be reasonably certain that this rarer species is also present on the landscape. With this heuristic, we were able to estimate the identification error rate attributable to mockingbirds. However, in our analysis, the identified mockingbirds did not mimick anything not previously detected in the acoustic survey. This is not to say that mockingbirds may not still serve an important role as sentinels for the presence of rare species, but just not in this study system.

We analyzed 137-hours across 70 transects for our initial acoustic analysis, and an additional 13.7-hours for our accuracy analysis, and analyzed a final 6.7-hours for our repeatability analysis of the Merlin Bird ID application. In the initial analysis we found that the Merlin Bird ID application had an average error rate of 18.73% ( Figure 1A ). Of that error, an average of 31.02% of the false positives were caused by mockingbirds ( Figure 1B ). For the accuracy analysis, we found that the average error rate was 29.17% ( Figure 1D ). The average false positive rate was 8.02% and the average false negative rate was 21.15% ( Figure 1D ). Of the false positive errors, an average of 26.68% were caused by mockingbirds ( Figure 1E ). In total, mockingbirds accounted for ~31% of the total false positive detections of 47 different species of bird in our surveys. Lastly, for the repeatability analysis, the Merlin Bird ID application provided identical species richness estimates among repeated surveys 91.3±3.13% of the time, with an average species richness per five minute focal survey of 5.7±1.8 species. However, there was greater viability in the species composition among surveys, which was only 75±22% similar. In addition, the most common three species detected during each repeated survey period was identical in 85±11% of the repeated surveys.

These findings show that while using the application Merlin Bird ID to detect bird assemblage at sites was reasonably accurate, and that accuracy can be improved upon with a simple heuristic scan of the data to remove false positives ( Figure 1G and Figure 1H). In the future, this heuristic can be adapted to provide a mechanism in which the Northern Mockingbird, and other mimicking species, can be distinguished between target species vocalization and eliminated from the resultant survey data to improve overall accuracy. Additionally, in the future with researchers utilizing bioacoustic surveys more often, data like this may influence the need for development an automated way to apply heuristic tools such as the one we used to rapidly clean acoustic datasets to account for mimicking species that may be imitating birds of interest. Lastly the results of our repeatability analysis identify an important caveat for any researchers wishing to rely on the Merlin bird identification application in lieu of trained field observers when conducting field studies of birds. Any single survey period is likely to contain several misidentifications even beyond those caused by mimics, and while the impact of this variation was minimal on overall estimate of species richness, it could have profound implications for characterizations of species assemblage at a location. We would recommend repeated surveys at a location and removal of outlier species from across survey attempts.
